# Prognostic significance of epigenetic regulatory gene expression in patients with non-small-cell lung cancer

**DOI:** 10.18632/aging.202600

**Published:** 2021-02-26

**Authors:** Zegui Tu, Xiancheng Chen, Tian Tian, Guo Chen, Meijuan Huang

**Affiliations:** 1 Department of Thoracic Oncology, West China Hospital of Sichuan University, Chengdu 610041, Sichuan, P.R. China; 2 West China Medical School, Sichuan University, Chengdu 610041, P.R. China; 3 State Key Laboratory of Biotherapy and Cancer Center, West China Hospital, Sichuan University, Chengdu 610041, Sichuan, P.R. China; 4 State Key Laboratory of Biotherapy/Collaborative Innovation Center for Biotherapy, West China Hospital, Sichuan University, Chengdu 610041, Sichuan, P.R. China; 5 Global Infotech Software Limited Corporation, Chengdu 610041, Sichuan, P.R. China

**Keywords:** epigenetic regulatory genes, non-small-cell lung cancer, expression patterns, prognosis

## Abstract

In this study, we used public databases to investigate the prognostic significance of epigenetic regulatory gene expression in patients with non small-cell lung cancer (NSCLC). Oncomine database analysis showed that the mRNA levels of seven epigenetic regulatory genes, *UHRF1, EZH2, TTF2, SUV39H2, PCNA, WHSC1* and *RAD54L*, genes were significantly upregulated in NSCLC patients as compared to normal lung tissues. Functional enrichment analysis of these seven genes showed that the most enriched GO terms were DNA repair and rhythmic process, whereas, the most enriched KEGG pathway was lysine degradation pathway. The mRNA and protein expression levels of UHRF1, EZH2, TTF2, WHSC1 and RAD54L significantly correlated with tumor stage in NSCLC patients. Moreover, NSCLC patients exhibiting higher UHRF1, EZH2, WHSC1 and RAD54L mRNA and protein expression levels had poorer progression-free survival and overall survival. These findings demonstrate that UHRF1, EZH2, WHSC1 and RAD54L are potential prognostic biomarkers to distinguish high-risk from low-risk NSCLC patients.

## INTRODUCTION

Lung cancer is more prevalent in the elderly, with individuals above 65 years accounting for nearly two-thirds of all new cases [1–5]. Non small-cell lung cancer (NSCLC) represents the most common histological type of lung cancer, accounting for nearly 85% of all cases, and includes lung adenocarcinoma (LUAD) and lung squamous cell carcinoma (LUSC) as the most common subtypes [6, 7]. Despite significant improvement in therapies over the past two decades, the overall survival rate for the majority of NSCLC patients is extremely low, especially for patients with metastasis [8]. There is thus an urgent need to identify novel biological markers that can serve as therapeutic targets and prognostic biomarkers for improving survival rates among patients with NSCLC.

Epigenetic alterations have emerged as major hallmarks of cancer [9]. Mutations in genes encoding chromatin modifying and chromatin remodeling proteins promote genome-wide DNA methylation changes in hematological malignancies and solid tumors [10–12]. DNA methylation changes induce chromatin decondensation, which significantly alters gene expression, promotes intra-tumor heterogeneity, and increases the invasiveness, drug resistance, and metastases of cancer cells [13–17]. Yang et al demonstrated that epigenetic regulatory genes such as *UHRF1, EZH2, TTF2, SUV39H2, PCNA, WHSC1*, and *RAD54L* were associated with alterations in genome-wide DNA methylation patterns in multiple cancer types [18]. Goto et al and Unoki et al reported that *UHRF1* was a potential diagnostic and prognostic biomarker of lung cancer [19, 20]. Daskalos et al demonstrated that *UHRF1* was associated with transcriptional silencing of multiple tumor suppressor genes by hypermethylating their promoters [21]. Despite these findings, the prognostic significance of *UHRF1* and other epigenetic regulatory genes has not yet been determined in non small-cell lung cancer (NSCLC). Therefore, in this study, we analyzed the prognostic significance of seven epigenetic regulatory genes, *UHRF1, EZH2, TTF2, SUV39H2, PCNA, WHSC1*, and *RAD54L*, in NSCLC patients.

## RESULTS

### Epigenetic regulatory genes are upregulated in NSCLC patient tissues

**Figure 1:**
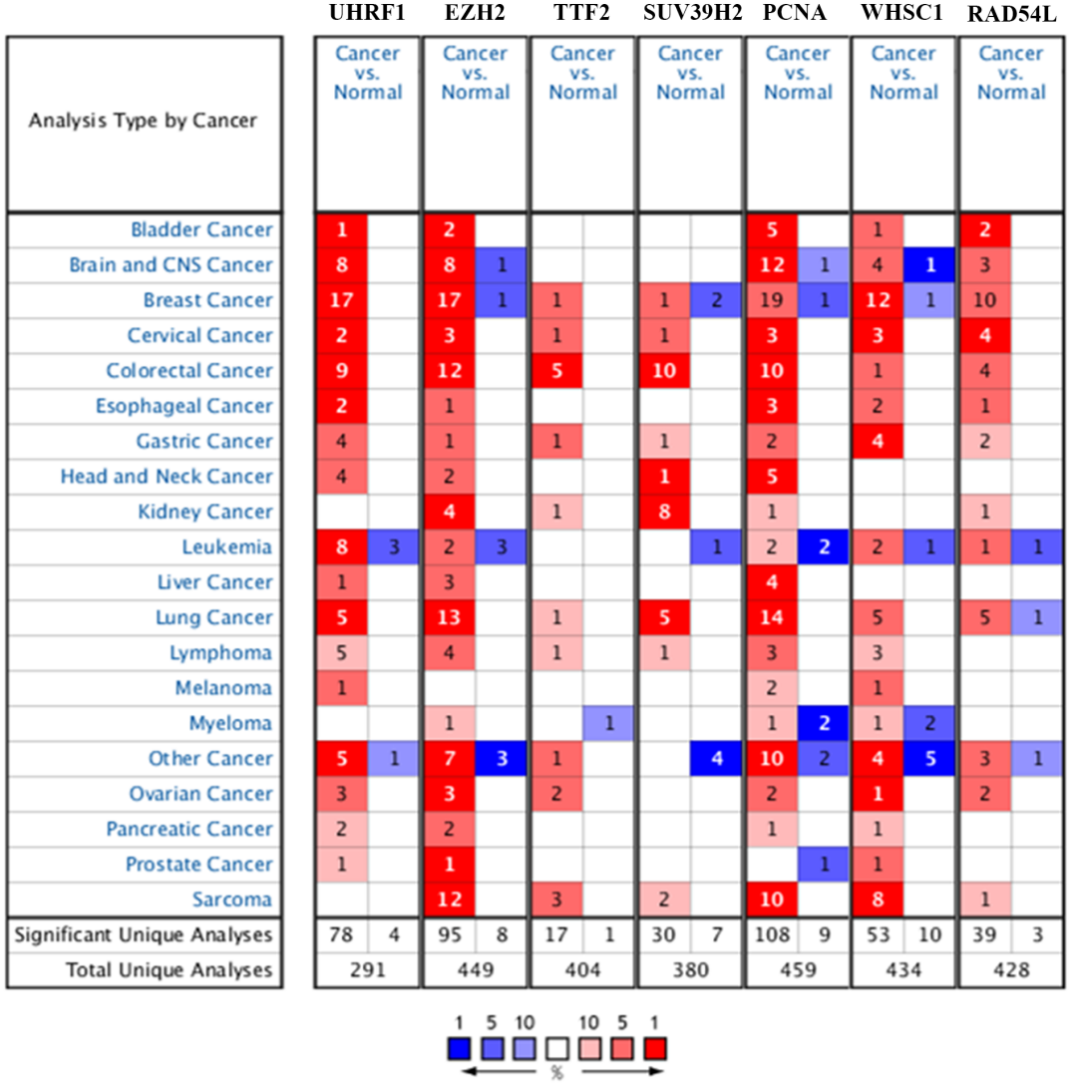
**Epigenetic regulatory gene expression in different types of cancers.** The expression levels of epigenetic regulatory genes in tumor and corresponding normal tissues from different cancer types in the Oncomine database. The differences between tumor tissues and corresponding normal tissues were compared using Student's t-test. Note: The cut-off values were p ≤ 0.01, fold change ≥ 2.0, and gene rank ≥ 10%. The mRNA levels of the seven epigenetic regulatory genes were higher in NSCLC tissues compared to the normal lung tissues.

The flow diagram of study strategy is shown in Supplementary Figure 1. Oncomine database (https://www.oncomine.org/) analysis showed that seven epigenetic regulatory genes, *UHRF1, EZH2, TTF2, SUV39H2, PCNA, WHSC1*, and *RAD54L,* were over-expressed in NSCLC tissues compared to the normal lung tissues in multiple NSCLC patient datasets (Figure 1 and Table 1).

**Table 1:** The differential epigenetic regulatory gene expression levels in NSCLC and normal lung tissue samples from the Oncomine database.

**Gene Symbol**	**Type Of NSCLC Versus Normal Lung Tissue**	**Fold Change**	**p Value**	**t Text**	**Number**	**Source and\or Reference**
UHRF1	Lung Adenocarcinoma	5.119	1.15E-18	11.96	110	Hou Lung Statistics
	Squamous Cell Lung Carcinoma	7.661	5.81E-23	17.491	92	Hou Lung Statistics
	Large Cell Lung Carcinoma	8.517	2.04E-08	8.462	84	Hou Lung Statistics
	Lung Adenocarcinoma	3.105	1.19E-20	13.705	116	Selamat Lung Statistics
	Lung Adenocarcinoma	2.81	5.94E-11	9.158	246	Okayama Lung Statistics
EZH2	Squamous Cell Lung Carcinoma	16.347	6.43E-08	6.577	38	Bhattacharjee Lung Statistics
	Lung Adenocarcinoma	3.455	4.92E-04	3.846	149	Bhattacharjee Lung Statistics
	Lung Adenocarcinoma	3.578	1.29E-08	6.588	57	Su Lung Statistics
	Squamous Cell Lung Carcinoma	3.041	3.83E-04	4.788	6	Garber Lung Statistics
	Large Cell Lung Carcinoma	2.549	4.00E-03	3.565	6	Garber Lung Statistics
	Squamous Cell Lung Carcinoma	6.474	4.45E-16	13.658	92	Hou Lung Statistics
	Lung Adenocarcinoma	3.189	2.29E-13	9.088	110	Hou Lung Statistics
	Large Cell Lung Carcinoma	6.418	1.65E-06	6.44	84	Hou Lung Statistics
	Lung Adenocarcinoma	2.017	3.15E-12	8.356	107	Landi Lung Statistics
	Lung Adenocarcinoma	2.554	6.52E-07	5.792	39	Stearman Lung Statistics
	Lung Adenocarcinoma	2.223	9.19E-13	10.145	246	Okayama Lung Statistics
TTF2	Large Cell Lung Carcinoma	2.064	5.80E-05	4.747	84	Hou Lung Statistics
SUV39H2	Large Cell Lung Carcinoma	3.479	7.63E-09	9.095	84	Hou Lung Statistics
	Lung Adenocarcinoma	2.056	1.12E-13	9.337	110	Hou Lung Statistics
	Squamous Cell Lung Carcinoma	2.476	1.67E-15	12.577	92	Hou Lung Statistics
	Large Cell Lung Carcinoma	2.435	8.00E-03	3.104	10	Garber Lung Statistics
PCNA	Lung Adenocarcinoma	2.92	4.05E-04	5.625	11	Yamagata Lung Statistics
	Squamous Cell Lung Carcinoma	2.86	7.72E-04	6.52	13	Yamagata Lung Statistics
	Large Cell Lung Carcinoma	3.082	6.00E-03	4.057	7	Yamagata Lung Statistics
	Squamous Cell Lung Carcinoma	19.337	1.62E-08	7.013	38	Bhattacharjee Lung Statistics
	Lung Adenocarcinoma	2.372	6.53E-06	6.338	46	Garber Lung Statistics
	Squamous Cell Lung Carcinoma	3.993	2.89E-05	5.452	19	Garber Lung Statistics
	Small Cell Lung Carcinoma	5.46	1.00E-03	6.696	10	Garber Lung Statistics
	Large Cell Lung Carcinoma	2.795	5.00E-03	4.306	10	Garber Lung Statistics
	Squamous Cell Lung Carcinoma	2.735	4.32E-04	7.35	10	Wachi Lung Statistics
	Lung Adenocarcinoma	2.322	1.39E-06	6.208	39	Stearman Lung Statistics
	Large Cell Lung Carcinoma	3.16	5.90E-08	7.989	84	Hou Lung Statistics
	Squamous Cell Lung Carcinoma	2.62	1.18E-12	10.612	92	Hou Lung Statistics
	Squamous Cell Lung Carcinoma	2.406	7.46E-09	6.577	62	Talbot Lung Statistics
WHSC1	Lung Adenocarcinoma	3.702	6.18E-07	5.589	57	Su Lung Statistics
	Squamous Cell Lung Carcinoma	2.146	8.13E-04	5.631	10	Wachi Lung Statistics
	Squamous Cell Lung Carcinoma	2.448	4.00E-03	2.864	38	Bhattacharjee Lung Statistics
	Large Cell Lung Carcinoma	2.913	7.87E-07	6.66	84	Hou Lung Statistics
RAD54L	Squamous Cell Lung Carcinoma	6.491	1.95E-05	4.778	38	Bhattacharjee Lung Statistics
	Squamous Cell Lung Carcinoma	2.832	1.59E-13	12.211	92	Hou Lung Statistics
	Large Cell Lung Carcinoma	3.442	3.52E-07	7.297	84	Hou Lung Statistics
	Lung Adenocarcinoma	4.994	7.42E-10	4.994	246	Okayama Lung Statistics

UHRF1 expression was significantly higher in lung adenocarcinoma (fold change=5.119, P=1.15E-18), squamous cell lung carcinoma (fold change=7.661; P=5.81E-23), and large cell lung carcinoma (fold change=8.517; P=2.04E-08) tissues in the Hou lung dataset. UHRF1 expression was also significantly higher in lung adenocarcinoma tissues in the Selamat lung dataset (fold change=3.105; P=1.19E-20) and Okayama lung dataset (fold change=2.81; P=5.94E-11), respectively.

EZH2 expression was significantly up-regulated in squamous cell lung carcinoma (fold change=16.347; P=6.43E-08) and lung adenocarcinoma (fold change=3.455; P=4.92E-04) tissues from the Bhattacharjee lung dataset, lung adenocarcinoma tissues (fold change=3.578; P=1.29E-08) from the Su lung dataset, squamous cell lung carcinoma (fold change=3.041; P=3.83E-04) and large cell lung carcinoma (fold change=2.549; P=4.00E-03) tissues from the Garber lung dataset, squamous cell lung carcinoma (fold change=6.474; P=4.45E-16), lung adenocarcinoma (fold change=3.189; P=2.29E-13) and large cell lung carcinoma (fold change=6.418; P=1.65E-06) tissues from the Hou lung dataset, and lung adenocarcinoma tissues from the Langi lung dataset (fold change=2.017; P=3.15E-12), Stearman lung dataset (fold change=2.554; P=6.52E-07), and Okayama lung dataset (fold change=2.223; P=9.19E-13), respectively.

TTF2 expression was significantly upregulated in large cell lung carcinoma tissues (fold change=2.064, p=5.80E-05) compared to the normal lung tissues in the Hou lung dataset.

SUV39H2 expression was significantly upregulated in large cell lung carcinoma (fold change=3.479; P=7.63E-09), lung adenocarcinoma (fold change: 2.056; P=1.12E-13), and squamous cell lung carcinoma (fold change: 2.476; P=1.67E-15) tissues compared to the normal lung tissues from the Hou lung dataset, and large cell lung carcinoma tissues (fold change=2.435; P=8.00E-03) from the Garber lung dataset.

PCNA was up-regulated in lung adenocarcinoma (fold change=2.92; P=4.05E-04), squamous cell lung carcinoma (fold change=2.86; P=7.72E-04), and large cell lung carcinoma (fold change=3.082; P=6.00E-03) tissues from the Yamagata lung dataset, squamous cell lung carcinoma tissues (fold=19.337; P=1.62E-08) from the Bhattacharjee lung dataset, lung adenocarcinoma (fold change =2.372; P=6.53E-06), squamous cell lung carcinoma (fold change=3.993; P=2.89E-05), and large cell lung carcinoma (fold change=2.795; P=5.00E-03) tissues from the Garber lung dataset, squamous cell lung carcinoma tissues (fold change=2.735; P=4.32E-04) from the Wachi lung dataset, lung adenocarcinoma tissues (fold change=2.322; P=1.39E-06) from the Stearman lung dataset, large cell lung carcinoma (fold change=3.16; P=5.90E-08) and squamous cell lung carcinoma (fold change=2.62; P=1.18E-12) tissues from the Hou lung dataset, and squamous cell lung carcinoma tissues (fold change=2.406; P=7.46E-09) from the Talbot lung dataset.

WHSC1 was significantly over-expressed in the squamous cell lung carcinoma tissues from the Wachi lung dataset (fold change=2.146; P=8.13E-04) and Bhattacharjee lung dataset (fold change=2.448; P=4.00E-03), lung adenocarcinoma tissues (fold change=3.702; P=6.18E-07) from the Su lung dataset, and large cell lung carcinoma tissues (fold change=2.913; P=7.87E-07) from the Hou lung dataset.

RAD54L was significantly over-expressed in squamous cell lung carcinoma tissues (fold change=6.491; P=1.95E-05) from the Bhattacharjee lung dataset, squamous cell lung carcinoma (fold change=2.832; P=1.59E-13) and large cell lung carcinoma (fold change=3.442; P=3.52E-07) tissues from the Hou lung dataset, and lung adenocarcinoma tissues (fold change=4.994; P=7.42E-10) from the Okayama lung dataset.

### Relationship between epigenetic regulatory gene expression and clinicopathological parameters of patients with NSCLC

**Figure 2:**
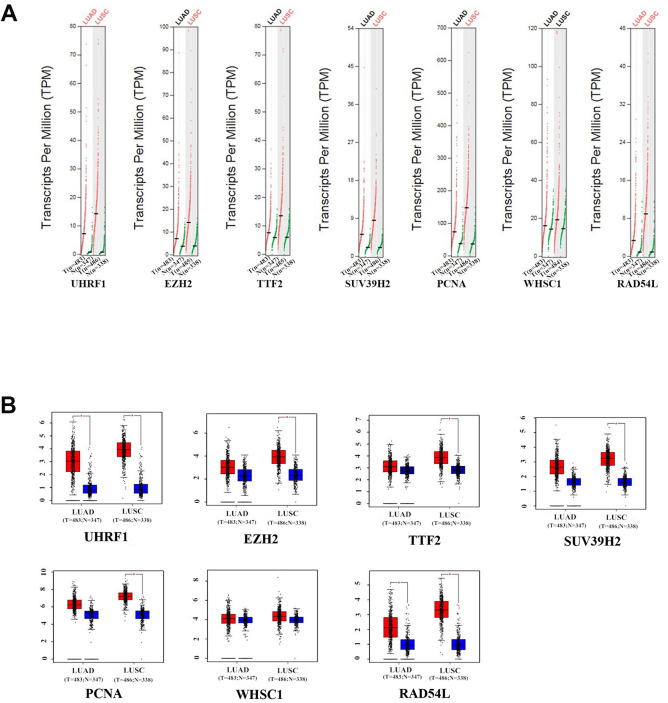
**The expression levels of the seven epigenetic regulatory genes in NSCLC tissues from the GEPIA database.** (**A**) Scatter diagram and (**B**) Box plots show the expression levels of the seven epigenetic regulatory genes in LUAD (Tumor:n=483;Normal:n=347) and LUSC (Tumor:n=486;Normal:n=338) tissues compared to the corresponding normal lung tissue samples.

**Figure 3:**
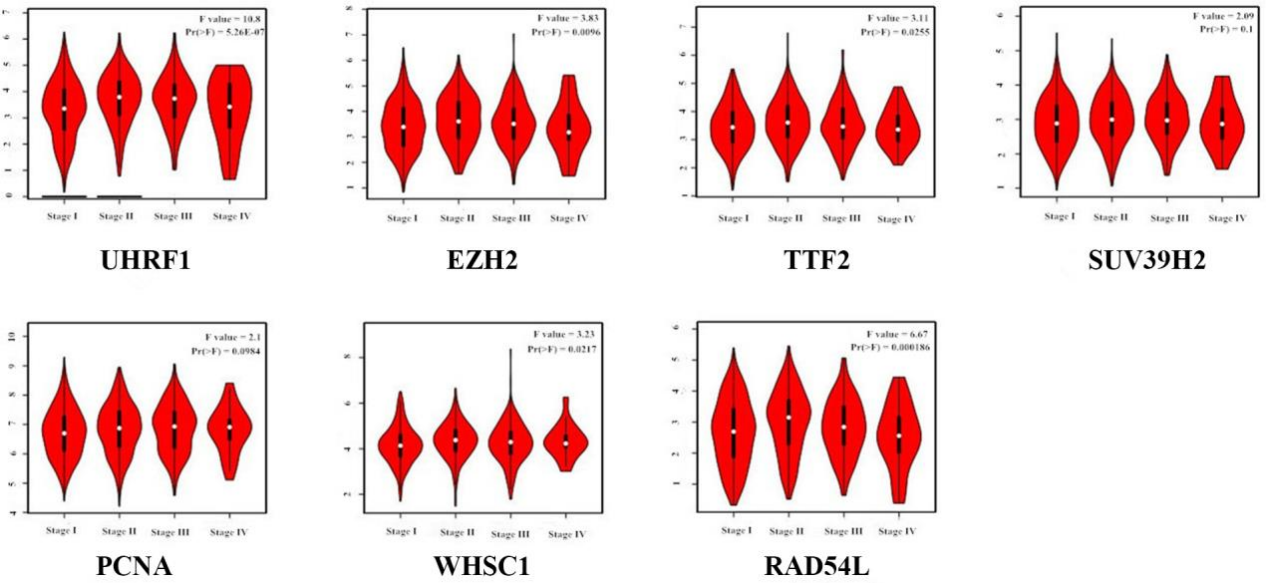
**Correlation analysis between epigenetic regulatory gene expression and tumor stages of NSCLC patients from the GEPIA database.** The expression levels of UHRF1, EZH2, TTF2, WHSC1 and RAD54L genes correlated significantly with the tumor stages of NSCLC patients. Note: P<0.05 was considered statistically significant.

GEPIA (Gene Expression Profiling Interactive Analysis) database (http://gepia.cancer-pku.cn/detail.php) analysis results showed that UHRF1, EZH2, TTF2, SUV39H2, PCNA, WHSC1 and RAD54L mRNA levels were significantly upregulated in LUAD and LUSC tissues compared to the normal lung tissues (Figure 2). Furthermore, expression levels of UHRF1, EZH2, TTF2, WHSC1 and RAD54L transcripts correlated significantly with the tumor stages of NSCLC patients (P<0.05; Figure 3). However, the expression levels of SUV39H2 and PCNA transcripts did not show any significant association with the tumor stages of NSCLC patients (P>0.05; Figure 3).

**Figure 4:**
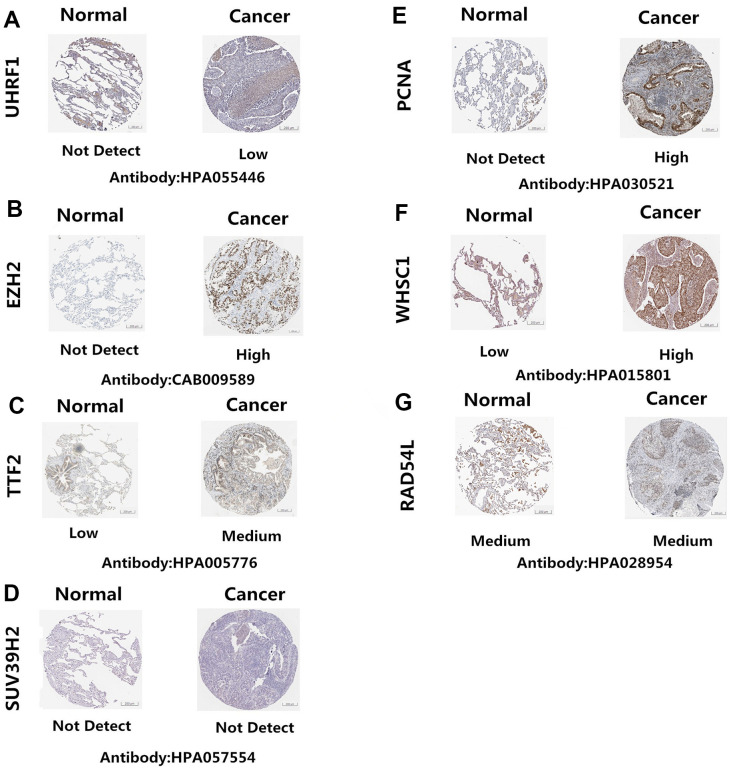
**Epigenetic regulatory protein expression in NSCLC and normal lung tissues.** The Human Protein Atlas database analysis results show the expression levels of UHRF1, EZH2, TTF2, PCNA, WHSC1, SUV39H2 and RAD54L proteins in NSCLC and normal lung tissues. UHRF1, EZH2, TTF2, PCNA, and WHSC1 protein levels were significantly upregulated in NSCLC tissues (**A**–**C**, **E**–**F**). SUV39H2 and RAD54L proteins were similar in the NSCLC and normal lung tissues (**D**, **G**).

The Human Protein Atlas (http://www.proteinatlas.org/) database analysis showed that UHRF1, EZH2, TTF2, PCNA, and WHSC1 protein levels were significantly upregulated in NSCLC tissues compared to the normal lung tissues (Figure 4A–4C, 4E, 4F). However, the expression levels of SUV39H2 and RAD54L proteins were similar in the NSCLC and normal lung tissues (Figure 4D, 4G)

### Prognostic significance of epigenetic regulatory gene expression in NSCLC patients

**Figure 5:**
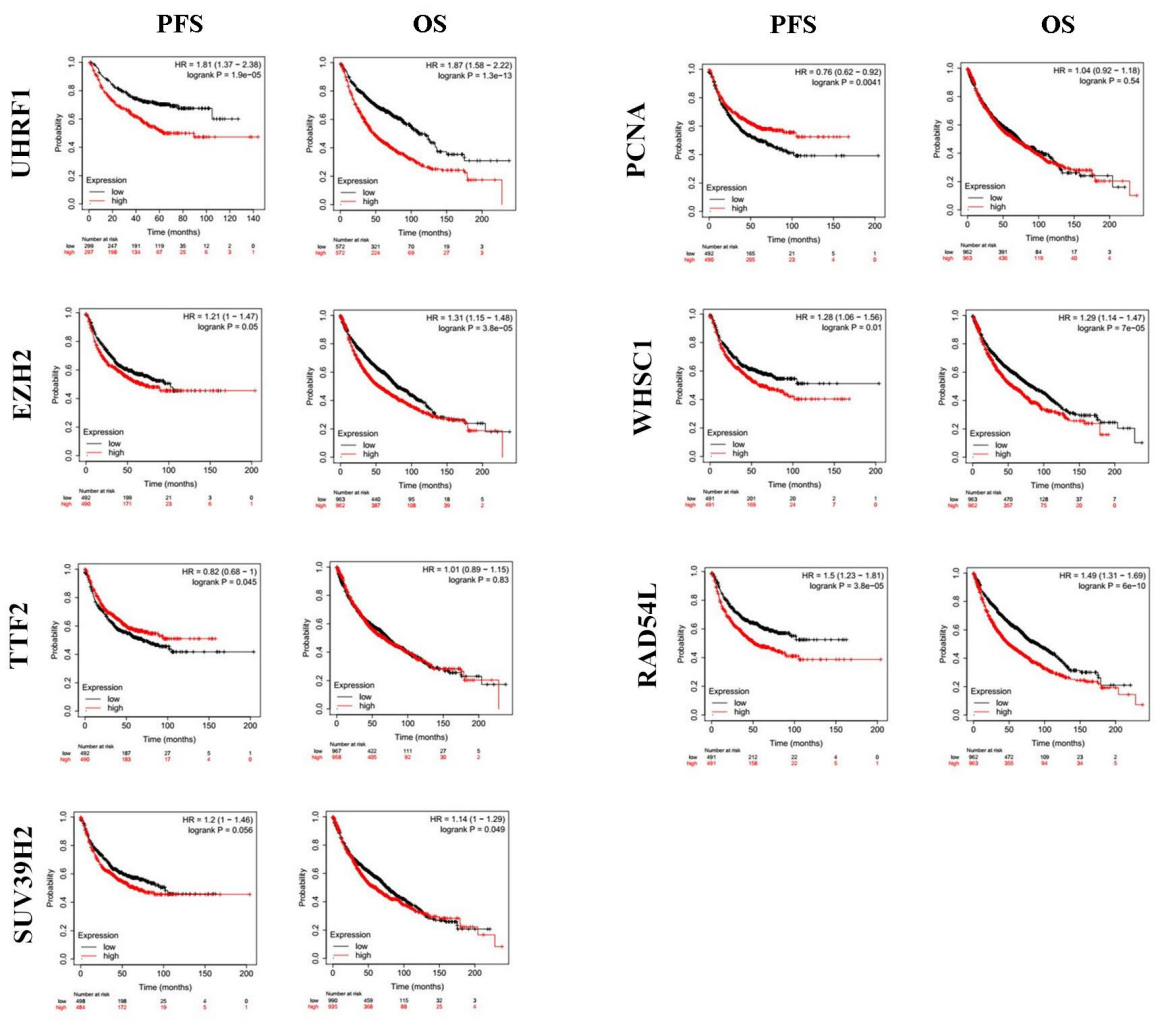
**The prognostic value of epigenetic regulatory gene expression in NSCLC tissues.** Kaplan-Meier survival curves and log-rank test shows that higher expression of UHRF1, EZH2, WHSC1 and RAD54L was significantly associated with worse progression-free survival (PFS) and overall survival (OS) of NSCLC patients.

**Figure 6:**
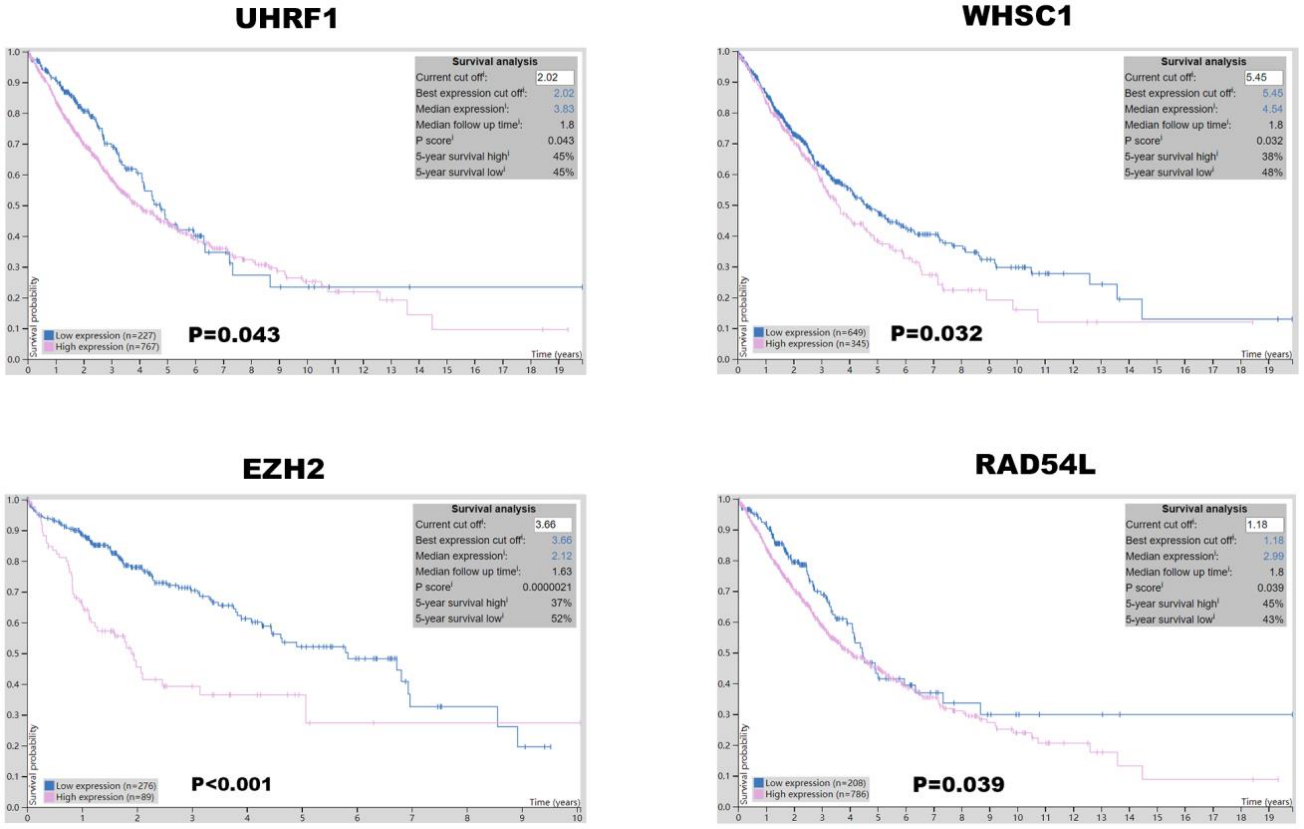
**Prognostic value of UHRF, EZH2, WHSC1, and RAD54L protein expression levels in NSCLC.** The Human Protein Atlas database analysis shows that the higher expression of UHRF1, EZH2, WHSC1 and RAD54L proteins was significantly associated with poorer prognosis of NSCLC patients.

Kaplan-Meier Plotter (https://kmplot.com/analysis/index.php?p=service&cancer=lung) database analysis showed that expression levels of the seven epigenetic regulatory genes were significantly associated with progression-free survival (PFS) and overall survival (OS) of NSCLC patients (Figure 5). Moreover, NSCLC patients with higher mRNA levels of UHRF1, EZH2, PCNA, WHSC1, and RAD54L were associated with significantly poorer PFS. NSCLC patients with higher TTF2 expression were associated with better PFS than those with lower TTF2 expression. Furthermore, NSCLC patients with higher mRNA expression levels of UHRF1, EZH2, SUV39H2, WHSC1 and RAD54L were associated with poorer OS rates. Overall, these results suggest that UHRF, EZH2, WHSC1, and RAD54L are potential prognostic biomarkers for patients with NSCLC. The Human Protein Atlas database analysis also showed that higher expression levels of UHRF, EZH2, WHSC1, and RAD54L proteins were associated with poorer prognosis in patients with NSCLC (Figure 6).

**Figure 7:**
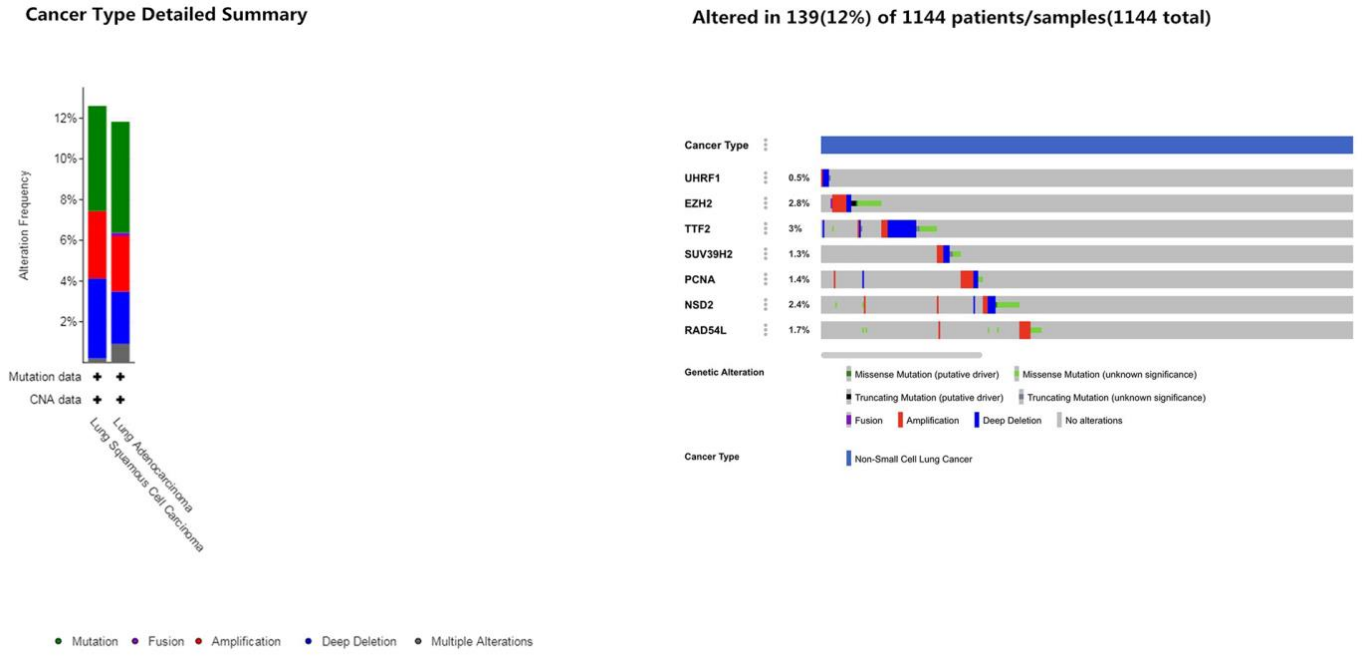
**Frequency of mutations in epigenetic regulatory genes in NSCLC samples.** The cBioportal database analysis shows that the frequency of mutations in *UHRF1*, *EZH2, TTF2, SUV39H2, PCNA, WHSC1/NSD2* and *RAD54L* were 0.5%, 2.8%, 3%, 1.3%, 1.4%, 2.4% and 1.7%, respectively, in NSCLC samples (n=1144).

**Figure 8:**
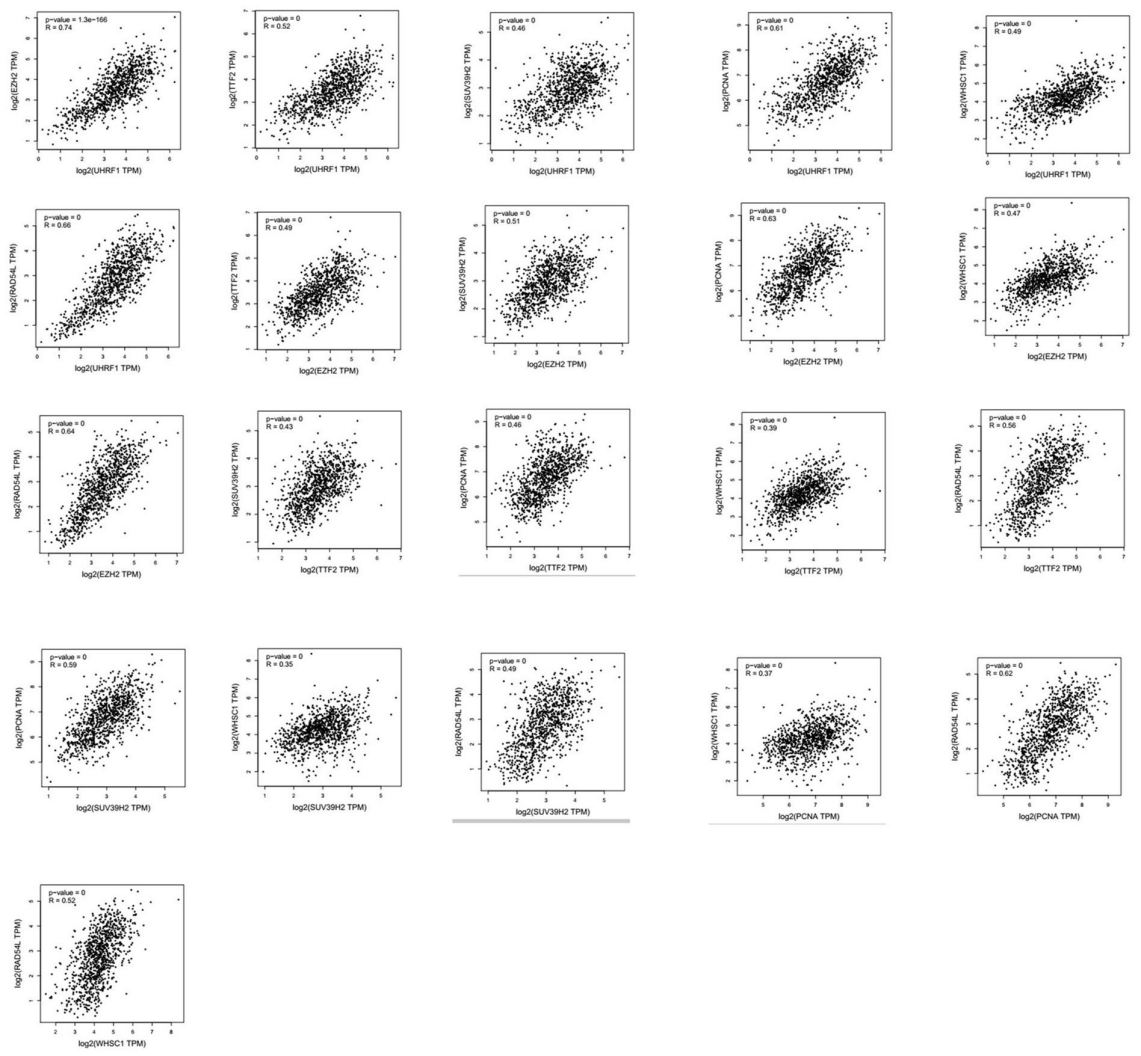
**Correlation analysis between epigenetic regulatory gene expression in NSCLC samples.** GEPIA database analysis shows positive correlation between different epigenetic regulatory genes.

Next, we analyzed alterations in the seven epigenetic regulatory genes in NSCLC tissues using the cBioPortal (Pan-Lung Cancer, The Cancer Genome Atlas, Nature genetics 2016; https://www.cbioportal.org/) database. The results showed alterations in *UHRF1, EZH2, TTF2, SUV39H2, PCNA, WHSC1/NSD2* and *RAD54L* genes with a frequency of 0.5%, 2.8%, 3%, 1.3%, 1.4%, 2.4% and 1.7%, respectively, in NSCLC patient samples (n=1144; Figure 7). Furthermore, GEPIA database analysis showed positive correlation between epigenetic regulatory gene expression. (Figure 8 and Table 2).

**Table 2:** Correlation between different epigenetic regulatory genes in NSCLC tissues.

1	0.74	0.52	0.46	0.61	0.49	0.66	UHRF1
0.74	1	0.49	0.51	0.63	0.47	0.64	EZH2
0.52	0.49	1	0.43	0.46	0.39	0.56	TTF2
0.46	0.51	0.43	1	0.59	0.35	0.49	SUV39H2
0.61	0.63	0.46	0.59	1	0.37	0.62	PCNA
0.49	0.47	0.39	0.35	0.37	1	0.52	WHSC1
0.66	0.64	0.56	0.49	0.62	0.52	1	RAD54L
UHRF1	EZH2	TTF2	SUV39H2	PCNA	WHSC1	RAD54L	

The correlation strength between different epigenetic regulatory genes is shown in the table. Note: very weak: 0.00-0.19; weak: 0.20-0.39; moderate: 0.40-0.59; strong: 0.60-0.7; very strong: 0.80-1.0.

### Functional enrichment analysis of potentially prognostic epigenetic regulatory genes in patients with NSCLC

**Figure 9:**
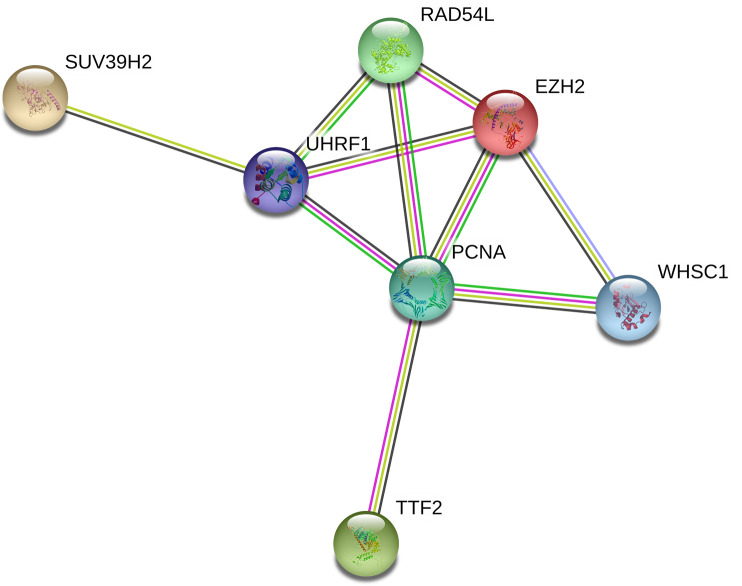
**Protein-protein interaction network between epigenetic regulatory genes in NSCLC.** The protein-protein interaction network based on known or predicted 3D structures of the seven epigenetic regulatory proteins shows co-expression relationships between RAD54L and EZH2, TTF2 and PCNA, as well as UHRF1 and PCNA.

Protein-protein interaction network analysis of the seven epigenetic regulatory genes and their co-expressing genes using String database (https://string-db.org/) showed co-expression of RAD54L and EZH2, TTF2 and PCNA, as well as UHRF1 and PCNA (Figure 9).

**Figure 10:**
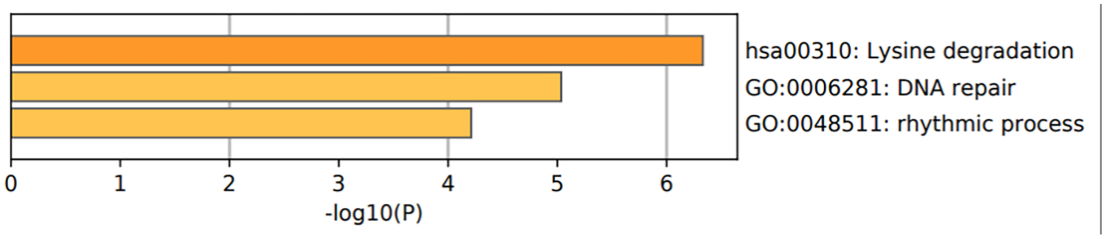
**Functional enrichment analysis of epigenetic regulatory genes.** The bar graph shows enriched GO terms and KEGG pathways related to the seven epigenetic regulatory genes. The p-values are shown according to the color scheme. The most enriched GO terms were DNA repair (GO:0006281) and rhythmic process (GO:0048511). The most enriched KEGG pathway was lysine degradation pathway.

We then performed the functional enrichment analysis of the epigenetic regulatory genes to determine significantly enriched gene ontology (GO) terms related to biological processes (BP), cellular components (CC), and molecular functions (MF) and Kyoto Encyclopedia of Genes and Genomes (KEGG) pathways using Metascape (https://metascape.org/gp/index.html). The results of GO analyses are shown in Figure 10 and Tables 3, 4. The most enriched GO terms for UHRF1 were positive regulation of DNA topoisomerase activity (GO:2000373), regulation of DNA topoisomerase activity (GO:2000371), positive regulation of isomerase activity (GO:0010912), nuclear heterochromatin (GO:0005720), euchromatin (GO:0000791), nuclear matrix (GO:0016363), hemi-methylated DNA-binding (GO:0044729), nucleosomal histone binding (GO: 0031493), and methyl-CpG binding (GO:0008327).

**Table 3:** Enriched biological functions and pathways related to the epigenetic regulatory genes.

**Gene Symbol**	**Biological Process (GO)**	**Cellular Component (GO)**	**Molecular Function (GO)**	**KEGG Pathway**
UHRF1	GO:2000373 positive regulation of DNA topoisomerase (ATP-hydrolyzing) activity;GO:2000371 regulation of DNA topoisomerase (ATP-hydrolyzing) activity;GO:0010912 positive regulation of isomerase activity	GO:0005720 nuclear heterochromatin;GO:0000791 euchromatin;GO:0016363 nuclear matrix	GO:0044729 hemi-methylated DNA-binding;GO:0031493 nucleosomal histone binding;GO:0008327 methyl-CpG binding	
EZH2	GO:0098532 histone H3-K27 trimethylation;GO:0014834 skeletal muscle satellite cell maintenance involved in skeletal muscle regeneration;GO:0036333 hepatocyte homeostasis	GO:0035098 ESC/E(Z) complex;GO:0045120 pronucleus;GO:0035097 histone methyltransferase complex	GO:0046976 histone methyltransferase activity (H3-K27 specific);GO:0000979 RNA polymerase II core promoter sequence-specific DNA binding;GO:0018024 histone-lysine N-methyltransferase activity	(hsa00310)Lysine degradation; (hsa05206)MicroRNAs in cancer
SUV39H2	GO:0036123 histone H3-K9 dimethylation;GO:0036124 histone H3-K9 trimethylation;GO:0051567 histone H3-K9 methylation	GO:0000775 chromosome, centromeric region;GO:0098687 chromosomal region;GO:0000785 chromatin	GO:0046974 histone methyltransferase activity (H3-K9 specific);GO:0018024 histone-lysine N-methyltransferase activity;GO:1904047 S-adenosyl-L-methionine binding	(hsa00310)Lysine degradation
PCNA	GO:0006977 DNA damage response, signal transduction by p53 class mediator resulting in cell cycle arrest;GO:1902990 mitotic telomere maintenance via semi-conservative replication;GO:0072431 signal transduction involved in mitotic G1 DNA damage checkpoint	GO:0043626 PCNA complex;GO:0070557 PCNA-p21 complex;GO:0044796 DNA polymerase processivity factor complex	GO:0000701 purine-specific mismatch base pair DNA N-glycosylase activity;GO:0032139 dinucleotide insertion or deletion binding;GO:0000700 mismatch base pair DNA N-glycosylase activity	(hsa03430)Mismatch repair; (hsa03410)Base excision repair; (hsa03030)DNA replication
WHSC1	GO:0048298 positive regulation of isotype switching to IgA isotypes;GO:0048296 regulation of isotype switching to IgA isotypes;GO:0048290 isotype switching to IgA isotypes	GO:0000785 chromatin;GO:0044427 chromosomal part;GO:0005694 chromosome	GO:0042799 histone methyltransferase activity (H4-K20 specific);GO:0046975 histone methyltransferase activity (H3-K36 specific);GO:0018024 histone-lysine N-methyltransferase activity	(hsa00310)Lysine degradation; (hsa05202)Transcriptional misregulation in cancer
RAD54L	GO:0000733 DNA strand renaturation;GO:0045003 double-strand break repair via synthesis-dependent strand annealing;GO:0007131 reciprocal meiotic recombination	GO:0005654 nucleoplasm;GO:0031981 nuclear lumen;GO:0070013 intracellular organelle lumen	GO:0015616 DNA translocase activity;GO:0036310 annealing helicase activity;GO:0097617 annealing activity	(hsa03440)Homologous recombination

**Table 4:** The top 3 clusters with the corresponding representative enriched term (one per cluster) related to the epigenetic regulatory genes.

**GO**	**Category**	**Description**	**Count**	**%**	**Log10(P)**	**Log10(q)**
hsa00310	KEGG Pathway	Lysine degradation	3	42.86	-6.33	-2.07
GO:0006281	GO Biological Processes	DNA repair	4	57.14	-5.03	-1.62
GO:0048511	GO Biological Processes	rhythmic process	3	42.86	-4.21	-1.01

“Count” is the number of epigenetic regulatory genes. “%” is the percentage of epigenetic regulatory genes that are found in the given ontology term. “Log10(P)” is the p-value in the log base 10. “Log10(q)” is the muti-test adjusted p-value in log base 10.

The top enriched GO terms related to EZH2 were histone H3-K27 tri-methylation (GO:0098532), skeletal muscle satellite cell maintenance involved in skeletal muscle regeneration (GO:0014834), hepatocyte homeostasis (GO:0036333), ESC/E(Z) complex (GO:0035098), pronucleus (GO:0045120), histone methyltransferase complex (GO:0035097), histone methyltransferase activity (H3-K27 specific) (GO:0046976), RNA polymerase II core promoter sequence-specific DNA binding (GO:0000979) and histone-lysine N-methyltransferase activity (GO:0018024).

The most enriched GO terms for SUV39H2 were histone H3-K9 dimethylation (GO:0036123), histone H3-K9 trimethylation (GO:0036124), histone H3-K9 methylation (GO:0051567), chromosome, centromeric region (GO:0000775), chromosomal region (GO:0098687), chromatin (GO:0000785), histone methyltransferase activity (H3-K9 specific) (GO:0046974), histone-lysine N-methyltransferase activity (GO:0018024) and S-adenosyl-L-methionine binding (GO:1904047).

The most enriched GO terms for PCNA were DNA damage response, signal transduction by p53 class mediator resulting in cell cycle arrest (GO:0006977), mitotic telomere maintenance via semi-conservative replication (GO:1902990), signal transduction involved in mitotic G1 DNA damage checkpoint (GO:0072431), PCNA complex (GO:0043626), PCNA-p21 complex (GO:0070557), DNA polymerase processivity factor complex (GO:0044796), purine-specific mismatch base pair DNA N-glycosylase activity (GO:0000701), dinucleotide insertion or deletion binding (GO:0032139) and mismatch base pair DNA N-glycosylase activity (GO:0000700).

The most enriched GO terms for WHSC1 were positive regulation of isotype switching to IgA isotypes (GO:0048298), regulation of isotype switching to IgA isotypes (GO:0048296), isotype switching to IgA isotypes (GO:0048290), chromatin (GO:0000785), chromosomal part (GO:0044427), chromosome (GO:0005694), histone methyltransferase activity (H4-K20 specific) (GO:0042799), histone methyltransferase activity (H3-K36 specific) (GO:0046975) and histone-lysine N-methyltransferase activity (GO:0018024).

The most enriched GO terms related to RAD54L were DNA strand renaturation (GO:0000733), double-strand break repair via synthesis-dependent strand annealing (GO:0045003), reciprocal meiotic recombination (GO:0007131), nucleoplasm (GO:0005654), nuclear lumen (GO:0031981), intracellular organelle lumen (GO:0070013), DNA translocase activity (GO:0015616), annealing helicase activity (GO:0036310), and annealing activity (GO:0097617) (Table 3).

Overall, the most enriched GO terms with the four potentially prognostic epigenetic regulatory genes were DNA repair (GO: 0006281) and rhythmic process (GO: 0048511) (Figure 10 and Table 4).

The most enriched KEGG pathways associated with EZH2 were lysine degradation (hsa00310) and microRNAs in cancer (hsa05206). lysine degradation (hsa00310) was the most enriched KEGG pathway associated with SUV39H2. The most enriched KEGG pathways associated with PCNA were mismatch repair (hsa03430), base excision repair (hsa03410) and DNA replication (hsa03030) pathways. The most enriched KEGG pathways associated with WHSC1 were lysine degradation (hsa00310) and transcriptional mis-regulation in cancer. Homologous recombination (hsa03440) pathway was associated with RAD54L (Table 3). Overall, three of the 4 prognostic associated epigenetic regulatory genes were associated with the lysine degradation pathway (Figure 10 and Table 4).

## DISCUSSION

Aberrant epigenetic regulation plays a critical role in tumorigenesis by altering genome-wide gene expression [22]. In this study, we comprehensively investigated the prognostic value of multiple epigenetic regulatory genes such as *UHRF1, EZH2, TTF2, SUV39H2, PCNA, WHSC1*, and *RAD54L* in NSCLC tissues using public databases.

*UHRF1* plays a significant role in skin, liver, prostate, breast, colon, kidney, bladder, and lung cancers, as well as hematological malignancies [21, 23–30]. UHRF1 is an epigenetic regulator that recruits DNMT1 and HDAC1 during cellular replication and modulates genome-wide DNA methylation levels [31, 32]. *UHRF1* is a potential driver gene that promotes aberrant DNA hyper-methylation in cancer cells [33]. UHRF1 targeted therapy sensitizes cancer cells to chemotherapeutic drugs by augmenting oxidative stress-mediated apoptosis [34]. In our study, UHRF1 expression was significantly upregulated in NSCLC tissues compared to the normal lung tissues. Moreover, UHRF1 expression correlated with the tumor stages of patients with NSCLC. Furthermore, higher UHRF1 expression was significantly associated with poor PFS and OS in NSCLC patients.

EZH2, the catalytic subunit of the polycomb-repressive complex 2 (PRC2), promotes epigenetic gene silencing through mono-, di-, tri-methylation of histone H3 at lysine 27 (H3K27) [35, 36]. EZH2 is transcriptionally induced by p53 and c-MYC, and post-transcriptionally regulated by several miRNAs and lncRNAs [37]. EZH2 expression correlates with poor prognosis of colorectal cancer, renal clear cell carcinoma, breast cancer, and bladder cancer [38–41]. In our study, EZH2 expression was significantly higher in NSCLC tissues compared to the normal lung tissues. Moreover, EZH2 expression correlated with the tumor stages of patients with NSCLC. EZH2 expression is associated with increased proliferation of several cancer cell lines [42–45]. Our study demonstrates that higher EZH2 expression correlates with poorer PFS and OS in NSCLC patients.

Thyroid transcription factor 2 (TTF2) is highly expressed in papillary thyroid cancer [46]. We demonstrated that TTF2 is overexpressed in NSCLC tissues compared to the normal lung tissues. Moreover, TTF2 expression correlates with the tumor stages of NSCLC patients. Furthermore, higher TTF2 expression significantly correlates with higher PFS rates in patients with NSCLC.

SUV39H2, also known as KMT1B, is a member of the SUV39 subfamily of lysine methyltransferases. It plays a significant role in di or tri-methylation of lysine 9 of histone H3 (H3K9), transcriptional regulation, and cell cycle [47, 48]. SUV39H2 is overexpressed in leukemia, lymphoma, breast cancer, colorectal cancer, gastric cancer, and lung cancer [49]. Zheng et al reported that SUV39H2 induced growth and progression of lung adenocarcinoma [50]. In this study, we demonstrated that SUV39H2 was overexpressed in NSCLC tissues compared to the normal lung tissues. However, SUV39H2 expression did not significantly correlate with the tumor stages of patients with NSCLC. Moreover, higher SUV39H2 expression was associated with worse OS in patients with NSCLC.

Proliferating cell nuclear antigen (PCNA) plays a significant role in cancer cell growth and survival by regulating DNA synthesis and repair [51]. Previous studies showed that higher expression of PCNA correlated with prognosis of colon adenocarcinoma, osteosarcoma, and gastric carcinoma [52–54]. Moreover, PCNA is a potential therapeutic target for NSCLC [55, 56]. In our study, PCNA was overexpressed in NSCLC tissues compared to the normal lung tissues. However, PCNA expression did not significantly correlate with tumor stages of patients with NSCLC. Higher PCNA expression was associated with poorer PFS in NSCLC patients.

WHSC1, a H3K36 methylation writer, is associated with leukemia, liver, endometrial and ovarian cancers [57–62]. Point mutations in WHSC1 promote genome-wide increase in H3K36me2 and H3K36me3 marks [59], which induces growth and progression of lung cancer [63]. In this study, we demonstrated that WHSC1 was overexpressed in NSCLC tissues compared to the normal tissues. Moreover, WHSC1 expression significantly associate with tumor stages of patients with NSCLC. Notably, higher WHSC1 expression significantly correlated with poorer PFS and OS in NSCLC patients.

DNA repair and recombination protein RAD54-like protein (RAD54L) is an oncogene involved in DNA repair and mitotic recombination. Higher expression of RAD54L correlates with poor prognosis of patients with glioblastoma and choroid plexus carcinoma [64, 65]. In this study, RAD54L expression was significantly higher in NSCLC tissues compared to the normal lung tissues. Moreover, RAD54L expression significantly correlated with tumor stages of patients with NSCLC. Furthermore, higher RAD54L expression significantly associated with poorer PFS and OS in NSCLC patients.

In conclusion, our study demonstrates that epigenetic regulatory genes such as *UHRF1, EZH2, TTF2, SUV39H2, PCNA, WHSC1*, and *RAD54L* are overexpressed in NSCLC tissues. Moreover, higher mRNA expression of UHRF1, EZH2, WHSC1 and RAD54L correlates with poorer PFS and OS of NSCLC patients. Hence, UHRF1, EZH2, WHSC1 and RAD54L are potential prognostic biomarkers for distinguishing high-risk from low-risk NSCLC patients.

## MATERIALS AND METHODS

### Oncomine database analysis

We analyzed 86733 samples from 715 gene expression array datasets in the Oncomine database (https://www.oncomine.org/) to determine the transcript levels of epigenetic regulatory genes in different cancers. The mRNA expression levels of epigenetic regulatory genes in the cancer specimens and normal tissues were compared using the Student’s t test. P < 0.01 and fold change ≥ 2.0 were used as threshold cutoff values to identify differentially expressed epigenetic regulatory genes in NSCLC tissues.

### GEPIA database analysis

We used the GEPIA interactive web-server (http://gepia.cancer-pku.cn/detail.php) [66] to analyze transcriptome data from 9736 NSCLC and 8587 normal lung tissue samples from the TCGA and GTEx projects. We analyzed the association between the expression of epigenetic regulatory genes and tumor stages of lung cancer patients (P<0.05). Spearman’s correlation coefficient was used to evaluate gene correlation.

### The Human Protein Altas database analysis

We used the Human Protein Atlas (http://www.proteinatlas.org/) open access database to analyze the expression levels of UHRF, EZH2, WHSC1, and RAD54L proteins in NSCLC and normal lung tissues. We also evaluated the correlation between the expression levels of the epigenetic regulatory proteins and overall survival of NSCLC patients. The univariate was controlled and P <0.05 was considered statistically significant.

### The Kaplan-Meier Plotter database analysis

We analyzed the association between the expression of epigenetic regulatory genes and the survival of lung cancer patients using the Kaplan-Meier Plotter database (https://kmplot.com/analysis/index.php?p=service&cancer=lung) [67]. The lung cancer patients were categorized into high- and low-expression groups based on the median gene expression and assessed using Kaplan-Meier survival curves. JetSet parameters were used to select the best set of epigenetic regulatory genes that are potential prognostic biomarkers after analyzing the Kaplan-Meier survival plots. We computed the hazard ratios (HR) with 95% confidence intervals and determined the P values using log-rank test. The univariate was controlled and P <0.05 was considered statistically significant.

### cBioPortal database analysis

The cBioPortal database (https://www.cbioportal.org/) was used to analyze the frequency of mutations and copy number alterations in the epigenetic regulatory genes in the lung cancer tissue samples from The Cancer Genome Atlas (Pan-Lung Cancer, TCGA, Nat Genet 2016) dataset.

### Protein-protein network construction using STRING database

We used the STRING database (https://string-db.org/) to construct a protein-protein interaction network between the seven differentially expressed epigenetic regulatory genes and their co-expressing genes in the lung cancer patients.

### Functional enrichment analysis using Metascape

We used Metascape database (https://metascape.org/gp/index.html) to perform functional enrichment analysis and identify enriched Gene Ontology (GO) terms related to biological processes(BP), cellular components(CC), and molecular functions(MF), and Kyoto Encyclopedia of Gene and Genomes (KEGG) pathways.

### Statistical analysis

The epigenetic regulatory genes were ranked after analyzing the transcriptome data from the Oncomine database according to fold changes and P-values were determined using the standard t-test. Spearman’s correlation analysis was used to determine the relationship of epigenetic regulatory genes and the association between mRNA expression of epigenetic regulatory genes and cancer stage using P<0.05 as the cut-off threshold in the GEPIA database. Kaplan-Meier survival curves were used to determine progression-free survival (PFS) and overall survival (OS) of NSCLC patients based on high- or low-expression of the seven epigenetic regulatory genes. Moreover, survival analysis was performed using the UHRF, EZH2, WHSC1, and RAD54L protein expression data from the NSCLC tissues in The Human Protein Altas database. P-value was determined for survival analysis based on a log-rank test and hazard ratios (HR) were determined.

## Supplementary Material

Supplementary Figure 1
