# Supplementary figures and images for: Prognostic significance of epigenetic regulatory gene expression in patients with non-small-cell lung cancer
Source: Aging (Albany NY). 2021 Feb 26;13(5):7397–415. doi: 10.18632/aging.202600 (PMC7993691; doi:10.18632/aging.202600)

## SUPPLEMENTARY FIGURE

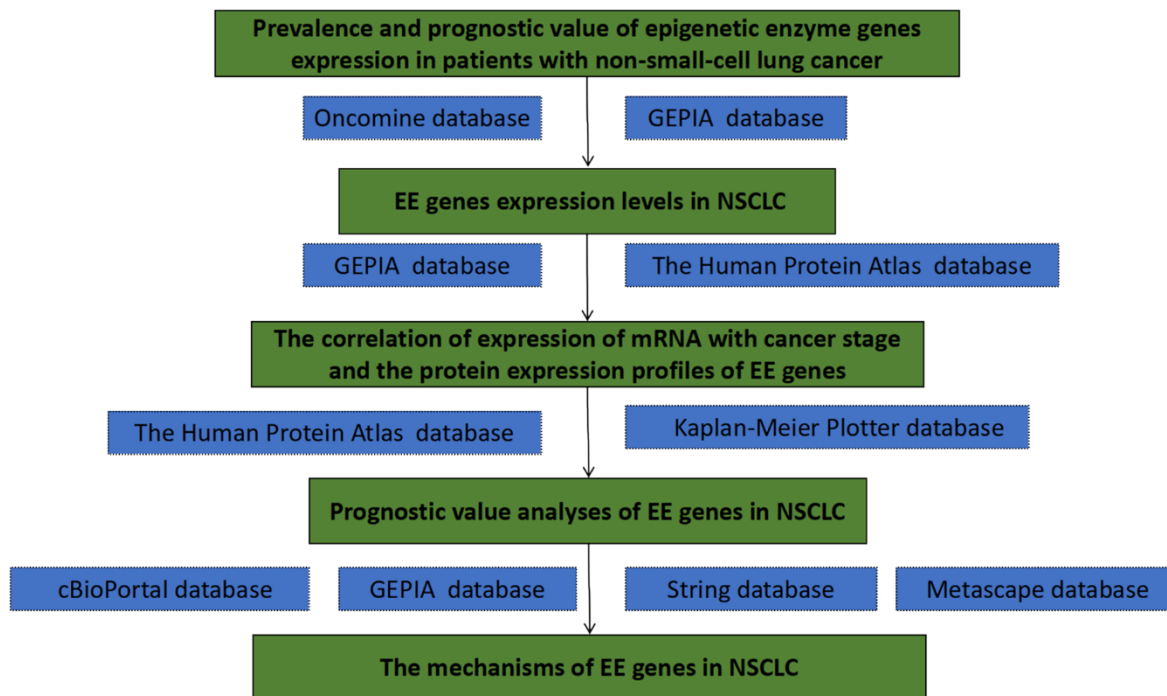

Supplementary Figure 1. The flow diagram of study strategy.

Supplement: Supplementary Figure 1 [file aging-13-202600-s001.pdf]
